# Virtual slides in peer reviewed, open access medical publication

**DOI:** 10.1186/1746-1596-6-124

**Published:** 2011-12-19

**Authors:** Klaus Kayser, Stephan Borkenfeld, Torsten Goldmann, Gian Kayser

**Affiliations:** 1 Institute of Pathology, Charite, Berlin, Germany; 2 IAT, Heidelberg, Germany; 3 Clinical and Experimental Pathology, Research Center Borstel, Borstel, Germany; 4 Institute of Pathology, University Freiburg, Freiburg, Germany

**Keywords:** Virtual slide, virtual microcopy, open access publication, image interpretation, image content information

## Abstract

**Background:**

Application of virtual slides (VS), the digitalization of complete glass slides, is in its infancy to be implemented in routine diagnostic surgical pathology and to issues that are related to tissue-based diagnosis, such as education and scientific publication.

**Approach:**

Electronic publication in Pathology offers new features of scientific communication in pathology that cannot be obtained by conventional paper based journals. Most of these features are based upon completely open or partly directed interaction between the reader and the system that distributes the article. One of these interactions can be applied to microscopic images allowing the reader to navigate and magnify the presented images. VS and interactive Virtual Microscopy (VM) are a tool to increase the scientific value of microscopic images.

**Technology and Performance:**

The open access journal Diagnostic Pathology http://www.diagnosticpathology.org has existed for about five years. It is a peer reviewed journal that publishes all types of scientific contributions, including original scientific work, case reports and review articles. In addition to digitized still images the authors of appropriate articles are requested to submit the underlying glass slides to an institution (DiagnomX.eu, and Leica.com) for digitalization and documentation. The images are stored in a separate image data bank which is adequately linked to the article. The normal review process is not involved. Both processes (peer review and VS acquisition) are performed contemporaneously in order to minimize a potential publication delay. VS are not provided with a DOI index (digital object identifier). The first articles that include VS were published in March 2011.

**Results and Perspectives:**

Several logistic constraints had to be overcome until the first articles including VS could be published. Step by step an automated acquisition and distribution system had to be implemented to the corresponding article. The acceptance of VS by the reader is high as well as by the authors. Of specific value are the increased confidence to and reputation of authors as well as the presented information to the reader. Additional associated functions such as access to author-owned related image collections, reader-controlled automated image measurements and image transformations are in preparation.

**Virtual Slides:**

The virtual slide(s) for this article can be found here: http://www.diagnosticpathology.diagnomx.eu/vs/1232133347629819.

## Introduction

Tissue based diagnosis or diagnostic surgical pathology is about to significantly change in its clinical importance and technology [1]. The times of autopsy and simple microscopic cellular morphology (H & E stained diagnosis) are still the gold standard for therapeutic decision making but are nowadays to a growing extent replaced by live imaging such as Computed Tomography (CT), nuclear resonance imaging (NRI), and nourished by molecular genetics and biology technology methods [2]. The combined application of these methods permits a so - called individualized diagnosis and treatment, especially for cancer patients [3,4]. The surgical pathologist acts as the clinician's guide, and the whole action is called predictive diagnosis [4,5].

In addition, modern communication tools and modules have been implemented in medicine, too [2,6-9]. These include fast communication lines (fiber optics), standardized communication tools such as digital imaging and communication in medicine (DICOM), picture archiving and communication system (PACS), or the internet. Hospital information systems (HIS) and laboratory information systems (LIS) have been implemented in nearly all larger hospitals and pathology institutes [10-12].

Digitalization of complete microscopic glass slides is an additional tool that fits into the digitized environment of an institute of pathology. Consecutively, approaches are ongoing to implement virtual microscopy (VM) into the pathologist's routine diagnostics [2,6,13-15].

Additional tools in this world of diagnosis include items that are mandatory for correct diagnostics, i.e., access to reference books, to experts who are specialized for certain diseases, or measurements of certain image features [16-19]. The performance of these aims is enhanced in electronic communication to a high degree.

In this article we describe a specialized tool that corresponds to VM and has been implemented in a specialized manner of electronic scientific communication, the so - called open access journals. The logistics and necessary implementation tools are described as well as the performance and acceptance of this innovative publication technology in medical sciences. In addition, the promising perspectives are briefly outlined.

### Journal data

**Figure 1:**
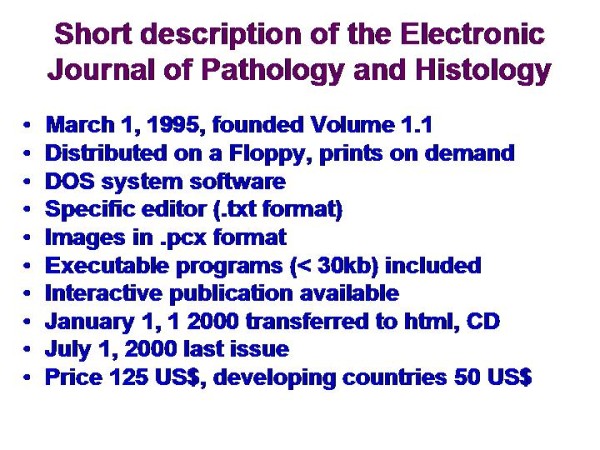
**Main characteristics of the first solely electronically distributed peer reviewed scientific journal in pathology (Electronic Journal of Pathology and Histology)**.

Open access publication has been developed after successful implementation of scientific electronic publication. In pathology, the Electronic Journal of Pathology and Histology (Elec J Pathol Histol) was the first scientific peer reviewed journal in pathology that has been solely electronically distributed to our knowledge [20]. It has been implemented in 1995 and was distributed via floppy-discs worldwide. Internet access and other electronic distribution media had not been available at that time. The main characteristics of Elec J Pathol Histol are displayed in <Figure 1>. Each floppy-disc was distributed with specific "reader" software, which also allowed the compilation and execution of programs. Tests of so - called interactive publication were also performed. These test allowed the reader to add own data to an already existing article, and to publish the "extended" version by including the authors of the previous article, after they had agreed. All images were displayed in a .pcx format. Although the peer reviewed and published articles had been cited worldwide, the application for inclusion into Reuter's citation index failed several times because a solely electronic distribution of scientific articles was not appreciated in the 1990s [21].

**Figure 2:**
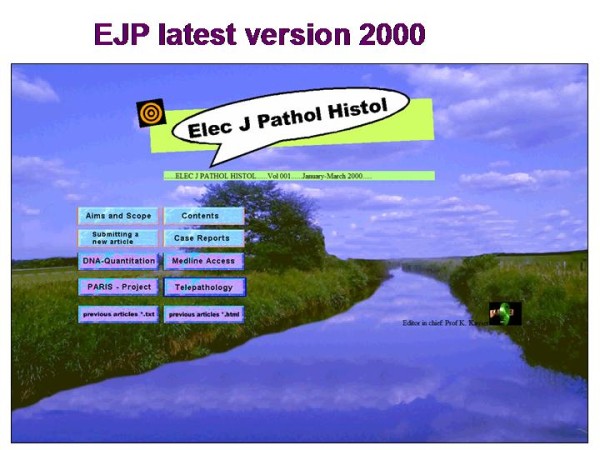
**View of the front page of the Electronic Journal of Pathology and Histology**.

The Elec J Pathol Histol was redesigned in HTLM format and distributed on a CD in 2000. However, several advantages of the DOS environment had to be given up (executable programs, interactive publication), and the production of the Elec J Pathol Histol was terminated in July 2000. The front page of last edition is depicted in <Figure 2>.

The Elec J Pathol Histol has been brought back to live in 2006 once the internet had been established and acknowledged as a useful and flexible communication medium. The new journal was named Journal of Diagnostic Pathology (Diagn Pathol), <http://www.diagnosticpathology.org>. The potential benefits of the internet embedding were analyzed, and corresponding mandatory formal changes were undertaken. These included:

Open access publication. The authors have to pay for the publication of their article. In compensation, they still inhabit the publication rights etc. of their article, and the readers have access to all published articles for free.

Authors who are working in developing countries can apply for waivers to publish their scientific results for free or at a remarkably reduced price.

The principle formal organization remains related to the conventional style of publication; i.e., Diagn Pathol still possesses its own "front page" and is not just a domain which might be embedded in another front page such as "medicine".

Access to and reading of the published articles is documented as well as the country of the reader, and statistically analyzed.

Reviewers are chosen from the editorial board and contemporary from authors who have published articles that are related to the content under request. These authors are chosen from articles cited in the National Institute of Health (NIH) library (PubMed).

The number of included color images, tables, or drawing etc. is not limited and free of additional charges.

All published articles are provided with an ID number (DOI) and indexed for interactive search of readers etc.

The introduction and establishment of Diagn Pathol lasted for about six months. The total number of published articles rose from 46 in 2006 to 83 in 2010, and will probably extent the level of 120 in 2011. The rejection rate increased from 30% in 2006 to > 50% in 2011. The journal possesses about 1,200 registered readers in 2011. Most articles are assessed by more than 500 readers within their first year of publication.

Diagn Pathol was included in the Citation Index (Reuter's) in 2010. It was calculated an impact factor of 1.39 for the year 2010.

### Including Virtual Slides in Open Access Scientific Publication

Virtual slides (VS) are the digital representation of completely digitized glass slides [2,13]. VS have been developed at the beginning of this century. Image acquisition machines (scanners) are commercially available from about 10 different companies (e.g. 3DHistech, Aperio, General Electric, Leica, Olympus, Phillips, and others). They are now in daily use for interdisciplinary conferences, teaching of medical students, and postgraduate education in most of the Medical Universities located in Western Europe and the United States. Some of the larger institutes of pathology are equipped with scanners that are embedded in the daily routine workflow of diagnosis, and several other institutes of pathology are investigating to replace the conventional workflow by virtual microscopy (VM) [2,6,10].

**Figure 3:**
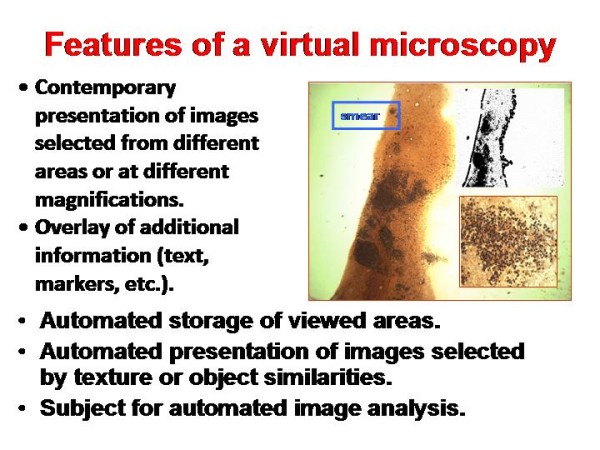
**Specific features of Virtual Microscopy (compared to conventional microscopy)**.

VS are comparable with conventional glass slides in image quality that has been demonstrated by several investigations [2,6,10]. The specific features of VM are summarized in <Figure 3>. In addition to the features of conventional microscopy they include contemporary viewing of different regions of interest (ROI) or stains, interactive labeling, automated scoring, and other electronic assistance.

**Figure 4:**
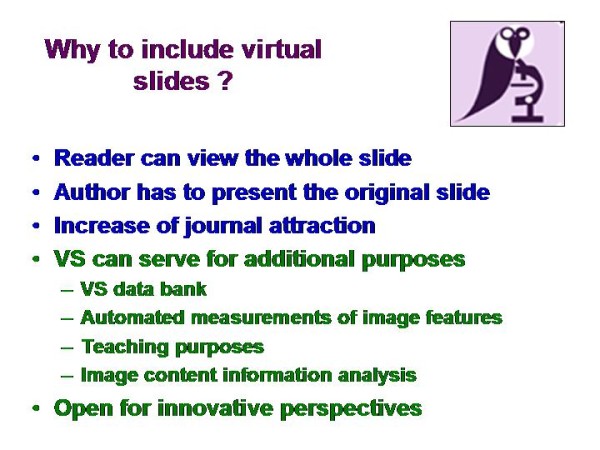
**Specific features of virtual slides focusing on open access publication**.

Including VS in an open access peer reviewed scientific journal includes several advantages for the authors, readers, and the publisher <Figure 4>:

The authors can demonstrate the morphological findings of whole microscopic image. The ROI can be analyzed independent from the authors' view.

The readers are assured for the originality of the findings, and can proof their own strategy for ROI selection.

The publisher increases the attraction of the included articles, and opens the journal for additional perspectives such as implementation of a repository or specific case collection, etc.

Constraints can be seen in the mandatory logistics:

The authors have to submit the original glass slides to a selected institution that scans the glass slides, because an official VS standard does not exist at present. Most of the companies have developed own specific viewers. A conversation of their own image format into a more general and open standard such as jp2000 is difficult if not impossible without knowing the individual image structure [22-27].

VS are large images of usually 2 - 3 GB in size. They usually require a specific viewer and a related image data bank that can handle these images. Thus, VS have to be organized in an electronic system that has to be separated from that of the published articles. In addition, the publication of still images should not be affected by VS.

After several trials, we decided to provide each article with dummy links that are kept empty if VS are not included in the article. These links are activated and connected to the corresponding images if VS are included.

**Figure 5:**
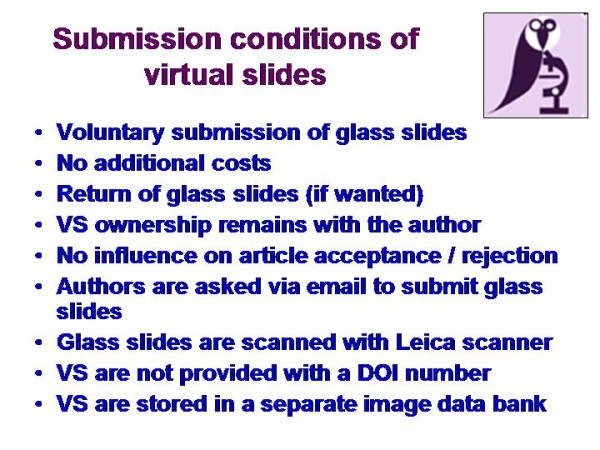
**Logistic frame of virtual slides to be published in open access journals**.

The chosen solution possesses logistic and content related advantages: The publication procedure of an article is not affected or delayed by VS as it is completely disconnected from the VS production, and VS do not require a DOI number (Digital Object Identifyer for article identification). Furthermore, VS can be included into each article at any time; even years after its publication by replacing the dummy link with an active one, and the reader has access to VS separately from included still images. The logistic frame is depicted in <Figure 5>. The reviewers are not informed about potential inclusion of VS in order to completely separate this issue from scientific considerations. Thus, VS do not involve, delay or promote the articles' publication.

### Experiences and Results

**Figure 6:**
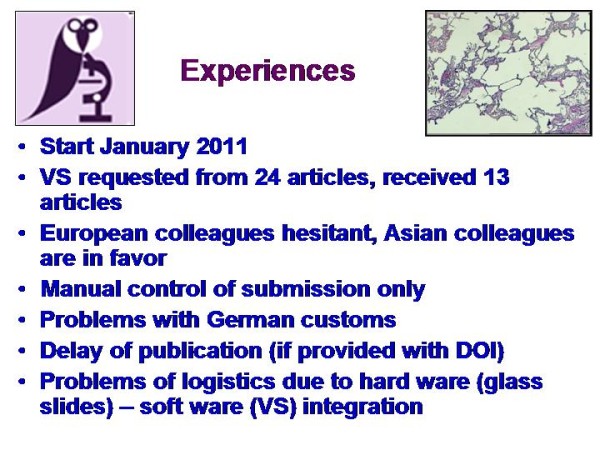
**Survey of published articles including virtual slides (Journal Diagnostic Pathology, September 2011)**.

The preparations of the described procedure lasted for about 6 months. The authors of newly submitted articles were informed via email about the VS technology and asked to submit the corresponding glass slides for digitalization. A summary of the authors' responses, number of included VS, and published articles is shown in <Figure 6 >. About 50% of requested authors submitted glass slides for VS publication, most of them are working in Asia. There was no time delay in peer reviewing and production process in comparison to articles that do not contain VS. VS seem to promote the interest of readers and the journals reputation as the number of submissions increased remarkably after publication of VS. However, also other reasons might play a significant role in this manner, such as fast review process, the citation index, or increased number of subscribers. The quality of all published VS was judged good or even very good. The access to and display of VS naturally depends on the network and included servers of the readers internet. The navigation and VM control is fast and reliable as only the viewing partitions of VS have to be downloaded.

## Discussion and Perspectives

This is the first report on successful VS publication in a peer reviewed scientific journal to our knowledge. The acceptance of this new technology by authors and readers can be judged good to very good. The reviewers are not informed about potential VS publication in the corresponding article. Thus, the reviewing process is completely separated from the newly introduced VS publication.

This might be a reason of debate. Reviewers are part of the scientific judgment of an article, and VS contribute to its scientific level too.

### Why to completely exclude reviewers from VS?

There are practical and theoretical reasons: One aim in electronic publication is to maintain a short review/production time which could be delayed in combining VS and review. In addition, the selection of published still images depends upon the author. Any errors in selecting additional correct ROI can be judged by the reader if VS are published too.

From the theoretical point of view, the diagnostic judgment in tissue based diagnosis can be distinguished in a) selection of the ROI, and b) the diagnostic statement [12,28-31]. In surgical pathology both algorithms are usually performed in only one combined step. Young colleagues are trained to perform the final diagnosis by viewing pre-selected areas (ROI) which are displayed in textbooks or atlases [2]. Publishing VS and still images independently from the review procedure separates these two steps, which is of additional educational significance.

The main constraint of the described method still remains in logistic "hardware" problems, especially in submission of requested glass slides. The mailing costs have to be covered by the authors. The glass slides have to be sent to an "image acquisition center" which controls the image quality. It is responsible to accurately document and handle VS. After several different trials to accurately link included still images with VS we decided to separately publish still images and VS. The separation of the "conventional" publication procedure from VS inclusion was favored by the production team. It could maintain the production speed. VS can be included at any time after publication. No intervention is needed. The provided links can be transferred to different servers at any time without involving the publication procedure of text, included images, and references. Thus, the journal diagnostic pathology has been "opened" to a new innovative distribution of image content information that offers new regions for additional applications.

Most of published VS are derived from case reports which usually describe rare diseases displaying educational significance. They are arranged in an appropriate data bank which will be transformed in an adequate repository in the next step. Certainly, the authors have to agree if the images will be included in such an electronic image retrieval system. Such a system will offer images of rare and educational significance combined with extensive clinical data such as the patient's history and development of the disease.

An additional potential application is related to quantitative object related measurements of VS [7,15]. They can be performed on a broad variety of stains including conventional and fluorescent immunohistochemistry using open access systems such as EAMUS™ or equivalent systems [7,15]. The reader can use these systems to select ROI from VS and to perform content related measurement.

In summary, the publication of VS in an open access peer reviewed scientific journal has been accepted by a broad community of pathologists and colleagues working in related medical and scientific fields shortly after its implementation. It significantly increases the reader's tools that can only be provided in an electronic communication environment such as individual selection of areas of interest, interactive measurements of specific objects, or image content information related selection of case reports for tissue based diagnosis and training.

## References

[B1] Goertler J Berghoff M Kayser G Kayser K Grid technology in tissue-based diagnosis: fundamentals and potential developments Diagn Pathol 2006 1 23 10.1186/1746-1596-1-23 16930477 PMC1564417

[B2] Kayser K Molnar B Weinstein RS Virtual Microscopy - Fundamentals - Applications - Perspectives of Electronic Tissue - based Diagnosis 2006 Berlin: VSV Interdisciplinary Medical Publishing

[B3] Marchio C Iravani M Natrajan R Lambros MB Savage K Tamber N Fenwick K Mackay A Senetta R Di Palma S Schmitt FC Bussolati G Ellis L Ashworth A Sapino A Reis-Filho JS Genomic and immunophenotypical characterization of pure micropapillary carcinomas of the breast J Pathol 2008 215 4 398 410 10.1002/path.2368 18484683

[B4] Comtesse N Keller A Diesinger I Bauer C Kayser K Huwer H Lenhof HP Meese E Frequent overexpression of the genes FXR1, CLAPM1 and EIF4G located on amplicon 3q26-27 in squamous cell carcinoma of the lung Int J Cancer 2007 120 12 2538 44 10.1002/ijc.22585 17290396

[B5] Nastase A Paslaru L Niculescu AM Ionescu M Dumitrascu T Herlea V Dima S Gheorghe C Lazar V Popescu I Prognostic and predictive potential molecular biomarkers in colon cancer Chirurgia (Bucur) 2011 106 2 177 85 21696062

[B6] Kayser K Gortler J Bogovac M Bogovac A Goldmann T Vollmer E Kayser G AI (artificial intelligence) in histopathology--from image analysis to automated diagnosis Folia Histochem Cytobiol 2009 47 3 355 61 10.2478/v10042-009-0087-y 20164018

[B7] Kayser K Hoshang SA Metze K Goldmann T Vollmer E Radziszowski D Kosjerina Z Mireskandari M Kayser G Texture- and object-related automated information analysis in histological still images of various organs Anal Quant Cytol Histol 2008 30 6 323 35 19160697

[B8] Kayser K Schultz H Goldmann T Görtler J Kayser G Vollmer E Theory of sampling and its application in tissue based diagnosis Diagnostic Pathology 2009 4 6 10.1186/1746-1596-4-6 19220904 PMC2649041

[B9] Kayser K Szymas J Weinstein RS Telepathology and Telemedicine - Communication, Electronic Education and Publication in e-Health 2005 Berlin: Veterinärspiegel Verlag

[B10] Rocha R Vassallo J Soares F Miller K Gobbi H Digital slides: present status of a tool for consultation, teaching, and quality control in pathology Pathol Res Pract 2009 205 11 735 41 10.1016/j.prp.2009.05.004 19501988

[B11] Weinstein RS Graham AR Richter LC Barker GP Krupinski EA Lopez AM Erps KA Bhattacharyya AK Yagi Y Gilbertson JR Overview of telepathology, virtual microscopy, and whole slide imaging: prospects for the future Hum Pathol 2009 40 8 1057 69 10.1016/j.humpath.2009.04.006 19552937

[B12] Wienert S Beil M Saeger K Hufnagl P Schrader T Integration and acceleration of virtual microscopy as the key to successful implementation into the routine diagnostic process Diagn Pathol 2009 4 3 10.1186/1746-1596-4-3 19134181 PMC2654030

[B13] Kayser G Görtler J Kluge N Wiech T Werner M Kayser K Standards in virtual microscopy: from tissue processing to image acquisition and visualization Diagnostic Pathology 2010 5 Suppl 1 S10 10.1186/1746-1596-5-S1-S10

[B14] Kayser K Borkenfeld S Görtler J Kayser G Image standardization in tissue - based diagnosis Diagnostic Pathology 2010 5 Suppl 1 S13 10.1186/1746-1596-5-S1-S13

[B15] Kayser K Radziszowski D Bzdyl P Sommer R Kayser G Towards an automated virtual slide screening: theoretical considerations and practical experiences of automated tissue-based virtual diagnosis to be implemented in the Internet Diagn Pathol 2006 1 1 10 10.1186/1746-1596-1-10 16764733 PMC1524814

[B16] Kayser K Basic considerations on DNA cytometry Elec J Pathol Histol 2002 8 021 08

[B17] Kayser K Dunnwald D Kazmierczak B Bullerdiek J Kaltner H Zick Y Andre S Gabius HJ Chromosomal aberrations, profiles of expression of growth-related markers including galectins and environmental hazards in relation to the incidence of chondroid pulmonary hamartomas Pathol Res Pract 2003 199 9 589 98 10.1078/0344-0338-00466 14621194

[B18] Kayser K Nwoye JO Kosjerina Z Goldmann T Vollmer E Kaltner H Andre S Gabius HJ Atypical adenomatous hyperplasia of lung: its incidence and analysis of clinical, glycohistochemical and structural features including newly defined growth regulators and vascularization Lung Cancer 2003 42 2 171 82 10.1016/S0169-5002(03)00289-7 14568684

[B19] Mireskandari M Kayser G Hufnagl P Schrader T Kayser K Teleconsultation in diagnostic pathology: experience from Iran and Germany with the use of two European telepathology servers J Telemed Telecare 2004 10 2 99 103 10.1258/135763304773391549 15068646

[B20] Kayser K Kayser G Bovin V Gabius HJ Quantitative Evaluation of Ligandohistochemistry with Cytoplasmic Markers: Program Structure and Application to Lung Carcinomas Elec J Pathol Histol 1995 1 4 954 02

[B21] Kayser K Introducing Diagnostic Pathology Diagnostic Pathology 2006 1 1 10.1186/1746-1596-1-1 PMC1475647 16759425

[B22] Ayad E Virtual telepathology in Egypt, applications of WSI in Cairo University Diagn Pathol 2011 6 Suppl 1 S1 10.1186/1746-1596-6-S1-S1 21489180 PMC3073202

[B23] Harnden P Coleman D Moss S Kodikara S Griffin NR Melia J Evaluation of the use of digital images for a national prostate core external quality assurance scheme Histopathology 59 4 703 9 10.1111/j.1365-2559.2011.03987.x 22014051

[B24] Mori I Ozaki T Taniguchi E Kakudo K Study of parameters in focus simulation functions of virtual slide Diagn Pathol 2011 6 Suppl 1 S24 10.1186/1746-1596-6-S1-S24 21489195 PMC3073218

[B25] Pantanowitz L Valenstein PN Evans AJ Kaplan KJ Pfeifer JD Wilbur DC Collins LC Colgan TJ Review of the current state of whole slide imaging in pathology J Pathol Inform 2011 2 36 10.4103/2153-3539.83746 21886892 PMC3162745

[B26] Roignot P Donzel JP Brunaud MD The use of virtual slides in the daily practice of a pathology laboratory Ann Pathol 2011 31 2 73 7 10.1016/j.annpat.2010.10.009 21601110

[B27] Tsuchihashi Y Expanding application of digital pathology in Japan--from education, telepathology to autodiagnosis Diagn Pathol 2011 6 Suppl 1 S19 10.1186/1746-1596-6-S1-S19 21489189 PMC3073212

[B28] Bubka A Bonato F Natural visual-field features enhance vection Perception 2010 39 5 627 35 10.1068/p6315 20677700

[B29] Jara-Lazaro AR Thamboo TP Teh M Tan PH Digital pathology: exploring its applications in diagnostic surgical pathology practice Pathology 2010 42 6 512 8 10.3109/00313025.2010.508787 20854068

[B30] Oger M Belhomme P Klossa J Michels JJ Elmoataz A Automated region of interest retrieval and classification using spectral analysis Diagn Pathol 2008 3 Suppl 1 S17 10.1186/1746-1596-3-S1-S17 18673505 PMC2500116

[B31] Rojo MG Gallardo AJ Gonzalez L Peces C Murillo C Gonzalez J Sacristan J Reading virtual slide using web viewers: results of subjective experience with three different solutions Diagn Pathol 2008 3 Suppl 1 S23 10.1186/1746-1596-3-S1-S23 18673512 PMC2500104

